# p-Cu_2_O-shell/n-TiO_2_-nanowire-core heterostucture photodiodes

**DOI:** 10.1186/1556-276X-6-575

**Published:** 2011-10-31

**Authors:** Tsung-Ying Tsai, Shoou-Jinn Chang, Ting-Jen Hsueh, Han-Ting Hsueh, Wen-Yin Weng, Cheng-Liang Hsu, Bau-Tong Dai

**Affiliations:** 1 Institute of Microelectronics and Department of Electrical Engineering, Center for Micro/Nano Science and Technology, Advanced Optoelectronic Technology Center, National Cheng Kung University, Tainan, 701, Taiwan, PR China; 2 National Nano Device Laboratories, Tainan, 741, Taiwan, PR China; 3 Department of Electronic Engineering, National University of Tainan, Tainan, 700, Taiwan, P. R. China

## Abstract

This study reports the deposition of cuprous oxide [Cu_2_O] onto titanium dioxide [TiO_2_] nanowires [NWs] prepared on TiO_2_/glass templates. The average length and average diameter of these thermally oxidized and evaporated TiO_2 _NWs are 0.1 to 0.4 μm and 30 to 100 nm, respectively. The deposited Cu_2_O fills gaps between the TiO_2 _NWs with good step coverage to form nanoshells surrounding the TiO_2 _cores. The p-Cu_2_O/n-TiO_2 _NW heterostructure exhibits a rectifying behavior with a sharp turn-on at approximately 0.9 V. Furthermore, the fabricated p-Cu_2_O-shell/n-TiO_2_-nanowire-core photodiodes exhibit reasonably large photocurrent-to-dark-current contrast ratios and fast responses.

## Introduction

UV photodetectors are important devices that have a range of commercial, research, and military applications. They can be used for space communication, ozone layer monitoring, and flame detection [1]. In recent years, high-performance GaN-based (including AlGaN and AlInN) [2-5], ZnO-based [6], and ZnSe-based [7] photodetectors have all been demonstrated. However, high-quality GaN-based UV photodetectors could only be prepared on a sapphire substrate, which is much more expensive as compared with a glass substrate. On the other hand, the photocurrent-to-dark-current contrast ratio of ZnO-based UV photodetectors is still low. Titanium dioxide [TiO_2_] is a potentially useful wide direct-bandgap material (3.2 eV for anatase and 3.0 eV for rutile) for UV photodetectors, solar cells, and gas sensors due to its outstanding physical, chemical, and optical properties [8-10]. TiO_2 _is a nontoxic naturally n-type semiconductor material which has a high-temperature stability and low-production costs.

For two-dimensional [2D] films, TiO_2 _UV photodetectors such as metal-semiconductor-metal detectors and Schottky barrier diodes have been demonstrated [11,12]. It is difficult to produce p- and n-type materials simultaneously, which is necessary for certain device applications. Zhang et al. reported the formation of a 2D TiO_2_/Cu_2_O composite film for a photocatalyst application using the metal ion-implantation method [13-15]. Cuprous oxide [Cu_2_O] is naturally a p-type direct-bandgap semiconductor with a cubic crystal structure and a room-temperature bandgap energy of 2.17 eV [16], which makes it ideal for TiO_2_-based p-n heterojunctions. Cu_2_O can be deposited using methods such as thermal oxidation, anodic oxidation, sputtering, solution growth, sol-gel, and electro-deposition [17-24]. Among these methods, sputtering is commonly used in the semiconductor industry. By carefully controlling the growth parameters, high-quality 2D Cu_2_O films can be produced by direct-current [DC] sputtering [18].

Recently, one-dimensional oxide semiconducting materials have attracted a lot of attention for potential application in optoelectronic devices due to their large surface-area-to-volume ratio [25]. Wu et al. reported the growth of TiO_2 _nanowires [NWs] on glass substrates by the thermal oxidation-evaporation method [26,27]. They produced single-crystalline TiO_2 _NWs, whose size and density were controlled by adjusting the growth parameters. However, no report on the fabrication of p-Cu_2_O-shell/n-TiO_2_-nanowire-core heterojunction UV photodetectors could be found in the literature, to our knowledge. The present study reports the deposition of p-Cu_2_O film onto n-TiO_2 _NWs by DC sputtering and the fabrication of radial p-Cu_2_O-shell/n-TiO_2_-nanowire-core photodiodes. The physical, electrical, and optical properties of the fabricated radial p-Cu_2_O-shell/n-TiO_2_-nanowire-core photodiodes are discussed.

## Experimental section

Before the growth of TiO_2 _NWs, a Corning 1737 glass substrate (Corning Display Technologies Taiwan Co., Ltd., Taipei City, Taiwan) was wet-cleaned with acetone and deionized water. The glass substrate was subsequently baked at 100°C for 10 min to evacuate moisture. A 400-nm-thick titanium [Ti] film layer was then deposited onto the glass substrate by electron-beam evaporation. Finally, the samples were annealed in a furnace at 700°C for 3 h to synthesize TiO_2 _NWs in argon [Ar] ambiance. The crystal quality of the as-grown NWs was then characterized by an X-ray diffractometer [XRD] (MXP 18, MAC Science Co., Tokyo, Japan). The surface morphology of the samples and the size distribution of the NWs were characterized by a field-emission scanning electron microscope [FE-SEM] (JEOL JSM-7000F, JEOL Ltd., Tokyo, Japan).

To investigate the deposition of Cu_2_O, glass was used as the substrate. The target used to deposit Cu_2_O was a 4-N pure copper block mounted on the cathode. The distance between the target and the sample was fixed at 60 mm. A rotating magnet fixed on the backside of the cathode was used to enhance the plasma bombardment effect. During sputtering, the Ar flow rate, deposition time, base pressure, and chamber pressure were kept at 15 sccm, 10 min, 2 × 10^-6 ^Torr, and 6 mTorr, respectively, and the DC power, O_2 _flow rate, and substrate temperature were 200 W, 4 sccm, and 25°C, respectively. The crystallography and structure of the deposited Cu_2_O and the Cu_2_O/TiO_2 _NWs were evaluated by XRD and FE-SEM.

**Figure 1:**
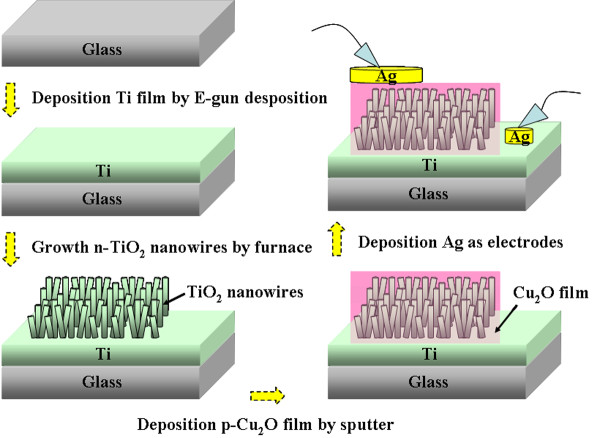
**Schematic diagram of fabricated p-Cu_2_O-shell/n-TiO_2_-nanowire-core for photodiode measurements**.

Prior to the fabrication of p-Cu_2_O-shell/n-TiO_2_-nanowire-core photodiodes, a small piece of glass was used to cover the TiO_2 _NWs to prevent the deposition of Cu_2_O in these regions. A 200-nm-thick Cu_2_O layer was subsequently deposited onto the TiO_2 _NWs. A 500-nm-thick silver layer was then sputtered onto the Cu_2_O layer and TiO_2 _NWs to serve as the p-electrode and n-electrode with a shadow mask. Figure 1 schematically shows the structure of the fabricated p-Cu_2_O-shell/n-TiO_2_-nanowire-core photodiodes. A picoammeter (HP-4145B semiconductor parameter analyzer, Agilent Technologies, Sta. Clara, CA, USA), connected via a GPIB controller to a computer, was then used to measure the current-voltage [I-V] characteristics of the fabricated diodes under darkness. The photo responses of the devices were also measured. During photo-response measurements, a 4-W mercury vapor lamp emitting at 365 nm was used as the excitation source.

## Results and discussion

**Figure 2:**
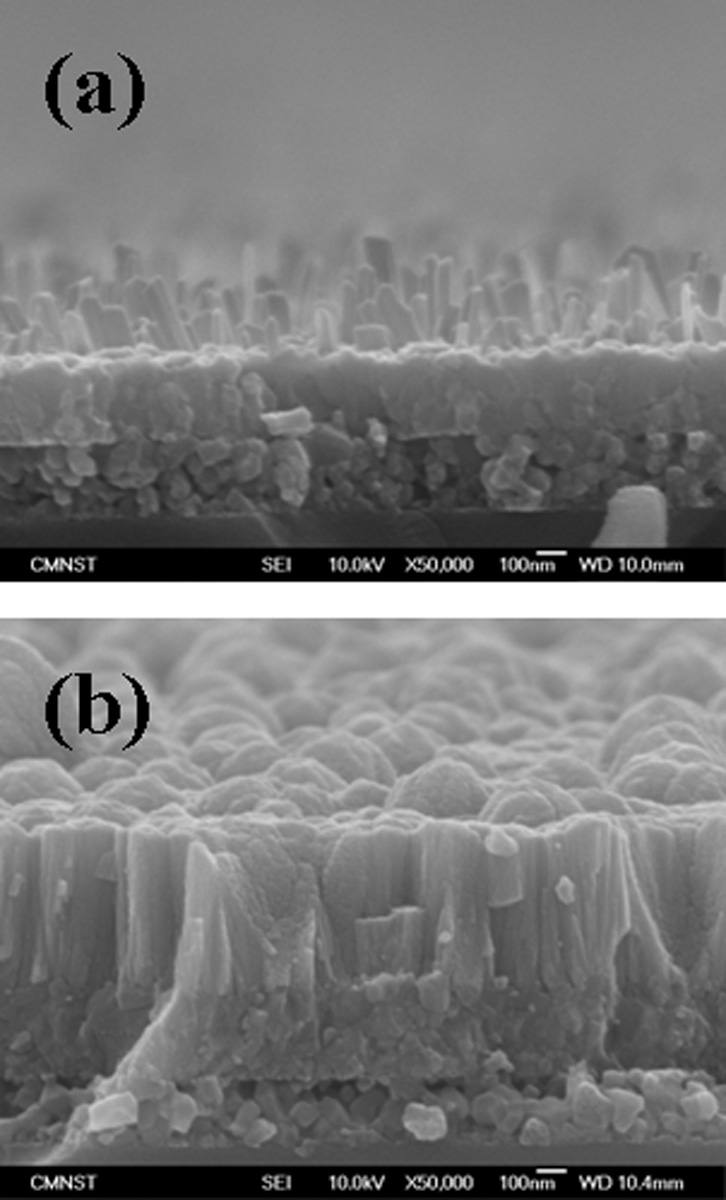
**Cross-sectional FE-SEM images**. (**a**) Pure TiO_2 _nanowires and (**b**) the sample with Cu_2_O deposited on TiO_2 _nanowires at 300°C with 200 W DC power, 6 mTorr chamber pressure, and 3 sccm O_2 _flow rate.

**Figure 3:**
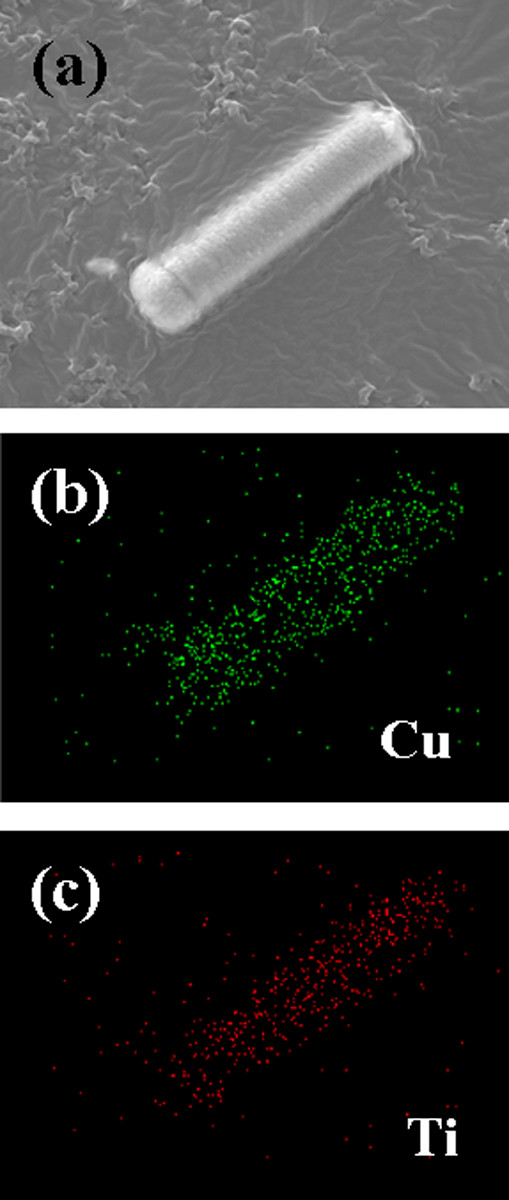
**FE-SEM and EDX images**. (**a**) An FE-SEM image of a single radial nanowire. EDX spectroscopic mapping images of (**b**) Cu and (**c**) Ti which were corresponded to (a).

Figure 2a shows a cross-sectional FE-SEM image of the TiO_2 _NWs prepared on a Ti/glass template. It can be clearly seen that high-density TiO_2 _NWs of various lengths were grown on the Ti/glass template. As shown in Figure 2a, it can be seen that the average length, diameter, and density of these TiO_2 _NWs were 0.3 μm, 50 nm, and 60 wires/μm^2^, respectively. Figure 2b shows a cross-sectional FE-SEM image of the sample with Cu_2_O deposited on TiO_2 _NWs. As shown, the deposited Cu_2_O filled the gaps between the TiO_2 _NWs with good step coverage to form radial Cu_2_O/TiO_2 _NWs. It was also found that the deposited Cu_2_O formed at the sample surface after filling the gaps. In order to investigate the coating performance of Cu_2_O, the deposited sample was scraped with tweezers into an alcohol solution, which was then ultrasonically treated for 20 min to disperse the NWs. The solution was dropped on carbon tape which was then placed on a hot plate to evacuate the alcohol. Figure 3a shows a SEM image of a single NW. Figures 3b and 3c show energy-dispersive X-ray [EDX] spectroscopic mapping images of Cu and Ti, respectively. These figures correspond to the SEM image shown in Figure 3a. After the deposition of Cu_2_O, Cu and Ti atoms were distributed over the entire NW. These results suggest that the sputtered Cu_2_O not only forms the head portion of the nanoclubs, but also forms nanoshells surrounding the TiO_2 _cores in the nanowire portion of the nanoclubs. The formation of such p-Cu_2_O-shell/n-TiO_2_-nanowire-core heterostructure should be able to provide us with a large junction area, which is important for the application of photodetectors.

**Figure 4:**
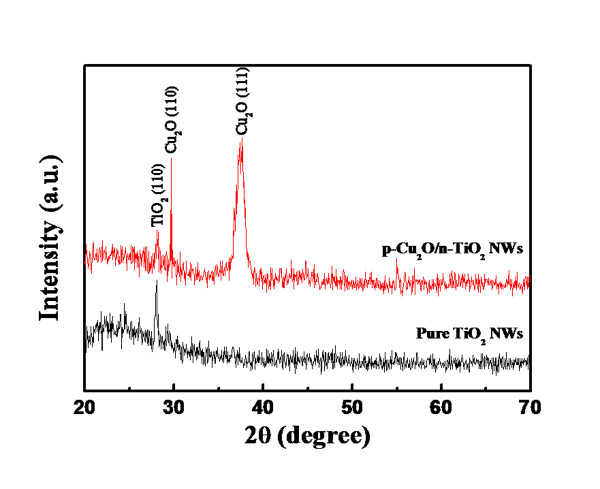
**XRD measurements of pure TiO_2 _nanowires and p-Cu_2_O/n-TiO_2 _nanowires obtained by DC sputtering**.

Figure 4 shows the crystallographic characteristics obtained from XRD measurements. For the pure TiO_2 _NWs used for adhesion, the peaks were attributed to the rutile-TiO2 (110) phase (JCPDS Card No. 88-1175). For the p-Cu_2_O/n-TiO_2 _NWs, the peaks were attributed to the (110) and (111) phases of the Cu_2_O phase (JCPDS Card No. 78-2076). No Ti-related signal was found, indicating that the Ti film changed into a TiO_2 _film after the annealing process.

**Figure 5:**
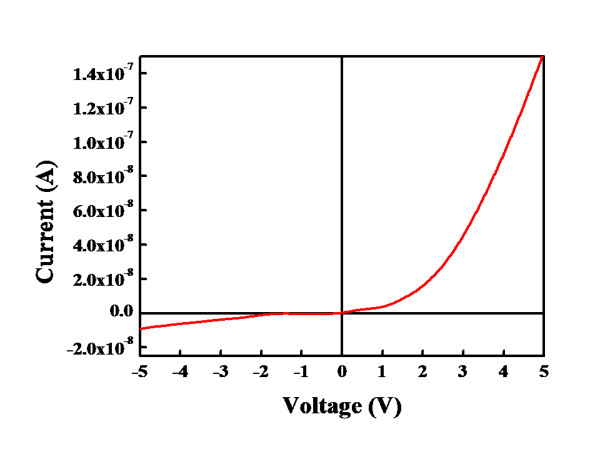
**Dark I-V characteristic measured from the fabricated radial p-Cu_2_O/n-TiO_2 _nanowires**.

Figure 5 shows the dark I-V characteristics measured from the fabricated radial Cu_2_O/TiO_2 _NWs. The rectifying behavior indicates that a p-n junction formed in the Cu_2_O/TiO_2 _NWs. The operation of the photodiode detector involves three steps (1) the generation of electron-hole [*e-h*] pairs by the absorption of incident light, whose photon energy exceeds the bandgap of the materials in the device; (2) the separation and transport of the *e-h *pairs by the internal electric field; and (3) the interaction of current with the external circuit to generate an output signal. Hence, the I-V characteristics of a photodiode in a dark environment are similar to those of a normal rectifying diode. If the p-n junction does not form, the generated *e-h *pairs will exhibit an ohmic character in the I-V curve and change the resistance. When a photodiode with a p-n junction is illuminated with optical radiation, the I-V characteristics shift according to the photocurrent and reverse current. The measured current in the photodiode, *I*_m_, is:

$$I_{\text{m}}=I_{\text{d}}-I_{\text{ph}}$$

where *I*_d _is the dark current and *I*_ph _is the photocurrent. The presence of a reverse current indicates that the photo response is due to the p-n junction, not the TiO_2 _NWs or the Cu_2_O. In the process of measurement under illumination, UV light passes through the TiO_2 _and illuminates the array of the radial p-Cu_2_O/n-TiO_2 _NWs; *e-h *pairs are produced in the radial NWs when the energy of the UV light is absorbed. The *e-h *pairs are separated by the internal electric field, and a photocurrent is simultaneously generated. Under forward bias, the turn-on occurred at approximately 0.9 V. With a +5-V applied bias, the forward current of the device was 1.53 × 10^-7 ^A, and with a -5-V applied bias, the reverse leakage current was 7.74 × 10^-9 ^A.

**Figure 6:**
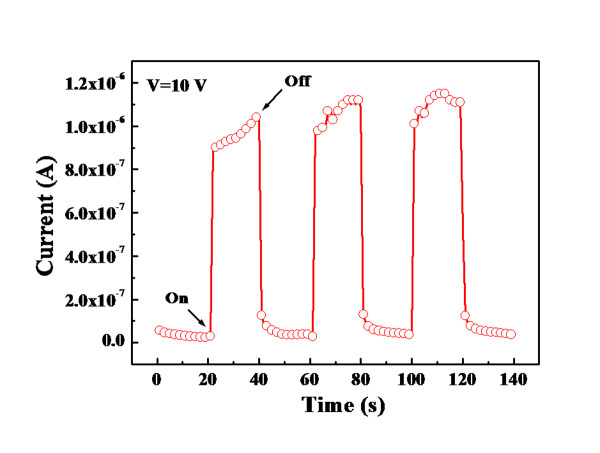
**Dynamic photo response measured from the fabricated p-Cu_2_O-shell/n-TiO_2_-nanowire-core photodiode**.

Figure 6 shows the dynamic photo response measured from the fabricated p-Cu_2_O-shell/n-TiO_2_-nanowire-core photodiode. With a +10-V applied bias, the dark reverse leakage current of the diode was only around 3.37 × 10^-8 ^A. However, the reverse leakage current increased rapidly to 1.15 × 10^-6 ^A upon UV illumination. When the UV lamp was turned off, the reverse leakage current rapidly decreased to its original value. The reasonably large photocurrent-to-dark-current contrast ratio and the fast responses suggest that the radial p-Cu_2_O-shell/n-TiO_2_-nanowire-core photodiodes proposed in this study are potentially useful for UV detector applications.

## Conclusions

The deposition of Cu_2_O onto well-aligned TiO_2 _NWs by DC sputtering was reported. With the proper sputtering parameters, the deposited Cu_2_O filled the gaps between the TiO_2 _NWs with good step coverage to form radial p-Cu_2_O/n-TiO_2 _NWs that exhibited rectifying I-V characteristics. The fabricated radial p-Cu_2_O-shell/n-TiO_2_-nanowire-core photodiodes had a reasonably large photocurrent-to-dark-current contrast ratio and fast responses.

## Competing interests

The authors declare that they have no competing interests.

## Authors' contributions

TYT carried out the nanowire experiments and data analysis and wrote the manuscript. SJC and TJH participated in data analysis and revised and finalized the manuscript. HTH designed the thin film and other experiments and data analysis. WYW participated in the revision of the manuscript. CLH provided the concept of the growth process of the nanowire. All the authors contributed to the preparation and revision of the manuscript and approved its final version.

## References

[B1] Monroy E Calle F Munoz E Omnes F Beaumont B Gibart P Visible-blindness in photoconductive and photovoltaic algan ultraviolet detectors J Electron Mater 1999 28 240 245 10.1007/s11664-999-0021-2

[B2] Chang SJ Ko TK Su YK Chiou YZ Chang CS Shei SC Sheu JK Lai WC Lin YC Chen WS Shen CF GaN-based p-i-n sensors with ITO contacts IEEE Sens J 2006 6 406 411

[B3] Zhang J Zhao H Tansu N Large optical gain AlGaN-delta-GaN quantum wells laser active regions in mid- and deep-ultraviolet spectral regimes Appl Phys Lett 2011 98 171111 10.1063/1.3583442

[B4] Zhang J Tong H Liu G Herbsommer JA Huang G Tansu N Characterizations of Seebeck coefficients and thermoelectric figures of merit for AlInN alloys with various In-contents J Appl Phys 2011 109 053706 10.1063/1.3553880

[B5] Zhang J Kutlu S Liu G Tansu N High-temperature characteristics of Seebeck coefficients for AlInN alloys grown by metalorganic vapor phase epitaxy J Appl Phys 2011 110 043710 10.1063/1.3624761

[B6] Weng WY Chang SJ Hsu CL Hsueh TJ A ZnO nanowire phototransistor prepared on glass substrates ACS Appl Mat Interfaces 2011 3 162 166 10.1021/am100746c 21226533

[B7] Lin TK Chang SJ Su YK Chiou YZ Wang CK Chang CM Huang BR ZnSe homoepitaxial MSM photodetectors with transparent ITO contact electrodes IEEE Trans Electron Devices 2005 52 121 123 10.1109/TED.2004.841288

[B8] Chiba Y Islam A Komiya R Koide N Han L Conversion efficiency of 10.8% by a dye-sensitized solar cell using a TiO_2 _electrode with high haze Appl Phys Lett 2006 88 223505 223507 10.1063/1.2208920

[B9] Kopidakis N Neale NR Zhu K Lagemaat JVD Frank AJ Spatial location of transport-limiting traps in TiO_2 _nanoparticle films in dye-sensitized solar cells Appl Phys Lett 2005 87 202106 202108 10.1063/1.2130723

[B10] Shen L Zhu G Guo W Tao C Zhang X Liu C Chen W Ruan S Zhong Z Performance improvement of TiO_2_/P3HT solar cells using CuPc as a sensitizer Appl Phys Lett 2008 92 073307 073309 10.1063/1.2884270

[B11] Xue H Kong X Liu Z Liu C Zhou J Chen W Ruan S Xu Q TiO_2 _based metal-semiconductor-metal ultraviolet photodetectors Appl Phys Lett 2007 90 201118 201120 10.1063/1.2741128

[B12] Kong X Liu C Dong W Zhang X Tao C Shen L Zhou J Fei Y Ruan S Metal-semiconductor-metal TiO_2 _ultraviolet detectors with Ni electrodes Appl Phys Lett 2009 94 123502 123504 10.1063/1.3103288

[B13] Zhang KJ Xu W Li XJ Zheng SJ Xu G Wang JH Photocatalytic oxidation activity of titanium dioxide film enhanced by Mn non-uniform doping Trans Nonferrous Met SOC China 2006 16 1069 1075 10.1016/S1003-6326(06)60379-8

[B14] Yasomanee JP Bandara J Multi-electron storage of photoenergy using Cu_2_O-TiO_2 _thin film photocatalyst Sol Energy Mat Sol Cells 2008 92 348 352 10.1016/j.solmat.2007.09.016

[B15] Zhang YG Ma LL Li JL Yu Y In situ Fenton reagent generated from TiO_2_/Cu_2_O composite film: a new way to utilize TiO_2 _under visible light irradiation Environ Sci Technol 2007 41 6264 6269 10.1021/es070345i 17937313

[B16] Siripala W Ivanovskaya A Jaramillo TF Baeck SH McFarland EW A Cu_2_O/TiO_2 _heterojunction thin film cathode for photoelectrocatalysis Sol Energy Mat Sol Cells 2003 77 229 237 10.1016/S0927-0248(02)00343-4

[B17] Ghijsen J Tjeng LH Elp JV Eskes H Westerink J Sawatzky GA Czyzyk MT Electronic structure of Cu_2_O and CuO Phys Rev B 1988 38 11322 11330 10.1103/PhysRevB.38.11322 9946011

[B18] Ishizuka S Kato S Okamoto Y Sakurai T Akimoto K Fujiwara N Kobayashi H Passivation of defects in polycrystalline Cu_2_O thin films by hydrogen or cyanide treatment Appl Surf Sci 2003 216 94 97 10.1016/S0169-4332(03)00485-9

[B19] Herion J Niekisch EA Scharl G Investigation of metal oxide/cuprous oxide heterojunction solar cells Sol Energy Mater 1980 4 101 112 10.1016/0165-1633(80)90022-2

[B20] Fortin E Masson D Photovoltaic effects in Cu_2_O-Cu solar cells grown by anodic oxidation Solid State Electron 1982 25 281 283 10.1016/0038-1101(82)90136-8

[B21] Fernando CAN Wetthasinghe SK Investigation of photoelectrochemical characteristics of n-type Cu_2_O films Sol Energy Mater Sol Cells 2000 63 299 308 10.1016/S0927-0248(00)00036-2

[B22] Armelao L Barreca D Bertapelle M Bottaro Y Sada C Tondello E A sol-gel approach to nanophasic copper oxide thin films Thin Solid Films 2003 442 48 52 10.1016/S0040-6090(03)00940-4

[B23] Golden TD Shumsky MG Zhou Y VanderWerf RA Leeuwen RAV Switzer JA Electrochemical deposition of copper (I) oxide films Chem Mater 1996 8 2499 2504 10.1021/cm9602095

[B24] Mahalingam T Chitra JSP Chu JP Sebastian PJ Preparation and microstructural studies of electrodeposited Cu_2_O thin films Mater Lett 2004 58 1802 1807 10.1016/j.matlet.2003.10.055

[B25] Hsueh TJ Chen HY Tsai TY Weng WY Yeh YM Dai BT Shieh JM Si nanowire-based photovoltaic devices prepared at various temperatures IEEE Electron Dev Lett 2010 31 1275 1277

[B26] Wu JM Shih HC Wu WT Electron field emission from single crystalline TiO_2 _nanowires prepared by thermal evaporation Chen Phys Lett 2005 413 490 494 10.1016/j.cplett.2005.07.113

[B27] Wu JM Shih HC Wu WT Formation and photoluminescence of single-crystalline rutile TiO_2 _nanowires synthesized by thermal evaporation Nanotechnology 2006 17 105 109 10.1088/0957-4484/17/1/017

